# Budd-Chiari syndrome in association with Behçet's disease: review of the literature

**DOI:** 10.1590/S1516-31802011000200009

**Published:** 2011-03-03

**Authors:** Daniela Carvalho, Fernando Oikawa, Nilce Mitiko Matsuda, Alice Tatsuko Yamada

**Affiliations:** I MD. Resident Physician, Hospital Municipal de Campo Limpo "Dr. Fernando Mauro Pires da Rocha", São Paulo, SP, Brazil.; II MD. Resident Physician, Hospital Municipal de Campo Limpo "Dr. Fernando Mauro Pires da Rocha", São Paulo, SP, Brazil.; III MD, PhD. Research associate, Department of Surgery and Anatomy, Faculdade de Medicina de Ribeirão Preto, Universidade de São Paulo (FMRP-USP), Ribeirão Preto, SP, Brazil.; IV MD, PhD. Cardiologist, Instituto do Coração (InCor), Hospital das Clínicas (HC), School of Medicine, Universidade de São Paulo (USP), São Paulo, and Hospital Municipal de Campo Limpo "Dr. Fernando Mauro Pires da Rocha", São Paulo, SP, Brazil.

**Keywords:** Budd-Chiari syndrome, Behçet's syndrome, Liver, Male, Young adult, Síndrome de Budd-Chiari, Síndrome de Behçet, Fígado, Masculino, Adulto jovem

## Abstract

The risk that patients with Behçet's disease may develop various thrombotic complications has been previously described. Although vascular complications from Budd-Chiari syndrome associated with Behçet's disease have been described, the pathogenic mechanisms are still unknown. Severe vascular complications present in Budd-Chiari syndrome associated with Behçet's disease are very common among young male adults. The objective of this study was to review the literature and present the association of Budd-Chiari syndrome with Behçet's disease.

## Abstract (pt)

O risco de pacientes com doença de Behçet desenvolverem várias complicações trombóticas já foi descrito. Apesar de complicações vasculares na síndrome de Budd-Chiari associada à doença de Behçet terem sido descritas, os mecanismos patogenéticos ainda são desconhecidos. Complicações vasculares graves presentes na síndrome de Budd-Chiari associada à doença de Behçet são muito mais comuns no adulto jovem do sexo masculino. O objetivo deste trabalho é o de revisar a literatura e apresentar a associação da síndrome de Budd-Chiari com a doença de Behçet.

## INTRODUCTION

Although the pathogenic mechanisms of vascular complications from Budd-Chiari syndrome in Behçet's disease are unknown, severe vascular complications from Budd-Chiari syndrome in patients with Behçet's disease are much more common in young adult male patients.^1–3^ Budd-Chiari syndrome associated with Behçet's disease has worldwide distribution, but it is more common in the Middle and Far East and rare in the Americas and Europe.^1–3^ Therefore, the purpose of this study was to review and summarize Budd-Chiari syndrome in association with Behçet's disease.

### Budd-Chiari syndrome

Budd-Chiari syndrome is caused by blood clots that completely or partially block the large veins that carry blood from the liver (hepatic veins) into the inferior vena cava.^4,5^ Some people have no overt symptoms, but others experience fatigue, abdominal pain, nausea, jaundice, enlarged liver and spleen, edema in the legs, ascites, and sometimes rupture and bleeding in the varicose veins of the esophagus. Usually, the symptoms develop gradually over weeks or months and Doppler ultrasonography can detect narrowed or blocked veins.^4,5^ Budd-Chiari syndrome is suspected when there are findings of an enlarged liver, ascites, liver failure or cirrhosis but there is no obvious cause, even after testing.^4,5^

Although the pathophysiology is unknown, the diagnosis of Budd-Chiari syndrome in patients with Behçet's disease is responsible for 3% of the cases of Budd-Chiari syndrome and the risk that patients with Behçet's disease will develop thrombotic complications is several times higher.^1–3^

### Behçet's disease

Behçet's disease is a multisystem disorder presenting with recurrent oral and/or genital ulcerations and chronic relapsing uveitis that may cause blindness and neurological impairments. The diagnosis is clinical since there is no specific evidence, pathognomonic symptoms or specific laboratory findings.^6–8^

According to the international criteria, the diagnosis of Behçet's disease requires the presence of recurrent oral ulceration in the absence of other clinical explanations, and two of the following; recurrent genital ulceration, eye lesions, skin lesions and/or a pathergy test.^6–8^ Although Behçet's disease has worldwide distribution, it is rare in the Americas and Europe and is more prevalent in Turkey, the Middle East and the Far East. It mainly affects young adults, and men have more severe vascular complications with this disease.^1,9–11^

### Budd-Chiari syndrome in association with Behçet's disease

Budd-Chiari syndrome or hepatic venous outflow obstruction was diagnosed in 30 patients over a 10-year period in a university hospital in Turkey and Behçet's disease constituted the largest group in the etiological distribution.^12^ Because of occlusion of the major hepatic veins, the adjacent inferior vena cava, or both, Budd-Chiari syndrome is a rare and serious complication of Behçet's disease. In the abovementioned cases series in Turkey, inferior vena cava involvement was more common in these patients.^12^

Budd-Chiari syndrome as a complication of Behçet's was seen in four young male patients in another clinical follow-up at the same Turkish university hospital.^13^ Out of 220 Tunisian patients who fulfilled the international criteria for the diagnosis of Behçet's disease, those with Budd-Chiari syndrome were selected. It was found that seven male patients with a mean age of 29 years who were already diagnosed with Behçet's disease had Budd-Chiari syndrome.^1^ Furthermore, the case of a young male patient with Behçet's syndrome and presenting with Budd-Chiari syndrome who died during emergency thrombectomy surgery was reported from another teaching hospital in Turkey.^10^

The evidence from countries in which Behçet's disease is prevalent suggests that this disease should be included among the diagnostic possibilities in cases of Budd-Chiari syndrome, since the third most common cause among a total of 75 patients diagnosed with Budd-Chiari syndrome was Behçet's disease.^14^

Thus, although Budd-Chiari syndrome associated with Behçet's disease has worldwide distribution, it is more common in the Middle and Far East and affects mainly young male adults.^1,10–13,15^ The association of Budd-Chiari syndrome and Behçet's disease in women is related to oral contraceptive usage and pregnancy.^16–18^

We performed a search for Budd-Chiari syndrome and Behçet's disease in relevant databases: Cochrane Database of Systematic Reviews, Embase Biomedical Answers, Literatura Latino-Americana e do Caribe em Ciências da Saúde (Lilacs) and the United States National Library of Medicine, National Institutes of Health (PubMed).^1,2,10–48^ The results are presented in **Table 1**.

**Table 1.** Databases for Budd-Chiari syndrome and Behçet's disease

Database	Search Strategy	Results		
		Articles found	Related articles	
Cochrane	Budd-Chiari syndrome and Behcet disease	0	0	-
Embase	Budd-Chiari syndrome and Behcet disease	71	38*	22 case reports 10 case series 2 prognostic studies 2 reviews 2 case-control studies
Lilacs	Budd-Chiari syndrome and Behcet disease	0	0	-
PubMed	Budd-Chiari syndrome and Behcet disease	45	37†	21 case reports 9 case series 3 prognostic studies 2 reviews 2 case-control studies

* References = ^1,2,10–45^;

† References = ^1,2,10–20,22–25,27,28,30–41,43–48^

Cochrane = Cochrane Database of Systematic Reviews; Embase = Embase Biomedical Answers; Lilacs = Literatura Latino-Americana e do Caribe em Ciências da Saúde; PubMed = United States National Library of Medicine, National Institutes of Health.

Although the mortality rate due to Behçet's disease is low, most patients with Behçet's disease who develop Budd-Chiari syndrome may die as a consequence of hepatic venous outflow obstruction.^9,10,13,15^ The hepatic venous outflow obstruction in Behçet's disease is often associated with other types of venous thrombosis and the prognosis may be favorable with medical interventions, including anticoagulation treatment for vasculitis and the use of diuretics when required.^11^

## CONCLUSION

Although the pathogenic mechanisms for Budd-Chiari syndrome are unknown, this vascular complication may be associated with Behçet's disease. In countries in which the prevalence of Behçet's disease is high, such as Turkey and others in the Middle and Far East, the evidence suggests that this disease should be included among the diagnostic possibilities in cases of Budd-Chiari syndrome. Severe vascular complications from Budd-Chiari syndrome in patients with Behçet's disease are much more common among young adult male patients.
